# Iron accumulation in macrophages promotes the formation of foam cells and development of atherosclerosis

**DOI:** 10.1186/s13578-020-00500-5

**Published:** 2020-11-26

**Authors:** Jing Cai, Meng Zhang, Yutong Liu, Huihui Li, Longcheng Shang, Tianze Xu, Zhipeng Chen, Fudi Wang, Tong Qiao, Kuanyu Li

**Affiliations:** 1 Department of Vascular Surgery, The Affiliated Drum Tower Hospital of Nanjing University Medical School, Nanjing, 210008 People’s Republic of China; 2 Department of General Surgery, The Affiliated Drum Tower Hospital of Nanjing University Medical School, Nanjing, 210008 People’s Republic of China; 3 Jiangsu Key Laboratory of Molecular Medicine, Medical School of Nanjing University, Nanjing, 210093 People’s Republic of China; 4 Department of Nutrition, School of Public Health, School of Medicine, Zhejiang University, Hangzhou, China

**Keywords:** Atherosclerosis, Iron metabolism, Fpn1, Macrophages, Foam cell formation

## Abstract

**Background:**

Macrophages that accumulate in atherosclerotic plaques contribute to progression of the lesions to more advanced and complex plaques. Although iron deposition was found in human atherosclerotic plaques, clinical and pre-clinical studies showed controversial results. Several epidemiological studies did not show the positive correlation between a systemic iron status and an incidence of cardiovascular diseases, suggesting that the iron involvement occurs locally, rather than systemically.

**Results:**

To determine the direct in vivo effect of iron accumulation in macrophages on the progression of atherosclerosis, we generated *Apoe*^*−/−*^ mice with a macrophage-specific ferroportin (*Fpn1*) deficiency (*Apoe*^*−/−*^*Fpn1*^*LysM/LysM*^). *Fpn1* deficiency in macrophages dramatically accelerated the progression of atherosclerosis in mice. Pathophysiological evidence showed elevated levels of reactive oxygen species, aggravated systemic inflammation, and altered plaque-lipid composition. Moreover, *Fpn1* deficiency in macrophages significantly inhibited the expression of ABC transporters (ABCA1 and ABCG1) by decreasing the expression of the transcription factor LXRα, which reduced cholesterol efflux and therefore promoted foam cell formation and enhanced plaque formation. Iron chelation relieved the symptoms moderately in vivo, but drastically ex vivo.

**Conclusions:**

Macrophage iron content in plaques is a critical factor in progression of atherosclerosis. The interaction of iron and lipid metabolism takes place in macrophage-rich atherosclerotic plaques. And we also suggest that altering intracellular iron levels in macrophages by systemic iron chelation or dietary iron restriction may be a potential supplementary strategy to limit or even regress the progression of atherosclerosis.

## Background

Atherosclerosis is the underlying cause of a majority of clinical cardiovascular events, including myocardial infarction, peripheral artery disease, stroke and coronary artery disease (CAD) [1]. Excessive fatty deposits and inflammatory cells accumulate during the formation and development of atherosclerotic lesions. As the major immune cells in atherosclerotic lesions, macrophages play a critical role in the development of atherosclerosis [2]. A central hallmark of atherosclerosis is foam cell formation characterized by uncontrolled lipoprotein accumulation within macrophages [3]. Despite decades of research, the molecular mechanisms underlying the uptake and efflux of lipids during this process remain to be fully understood [4].

Iron is an essential element for many biological processes, such as DNA repair, cellular respiration, oxidation and reduction reactions, and oxygen transport. In 1981, Sullivan initially found a correlation between atherosclerosis and iron deposition, called the “iron hypothesis”, which proposes that iron overload promotes cardiovascular diseases, while iron deficiency protects against ischemic heart disease [5, 6]. Interestingly, several epidemiological studies found that a high iron status was not associated with an increased incidence of CAD in humans; in contrast, an elevated status was correlated with a reduced CAD risk [7, 8]. Although the iron concentration is higher in human atherosclerotic plaques than in healthy arterial tissue [9], it is still unclear whether iron accumulation in atherosclerotic plaques is a cause or a consequence, whether iron deposition is deleterious, and whether the associated harmful effects are cell specific [10]. Considering the key roles of macrophages in the formation and progression of atherosclerotic plaques, combined with the fact that macrophages provide a large amount of iron in the circulation to meet systemic requirements by recycling iron from senescent red blood cells [11], selective iron deposition in macrophages has been proposed as a mechanism underlying accelerated atherosclerosis progression via catalytic generation of reactive oxygen species (ROS) and thus promotion of foam cell formation [12, 13]. Several mouse models of iron overload (i.e., high-iron diet or injection with iron-dextran [14, 15] and hereditary hemochromatosis (HH) [16–18] characterized by systemic iron overload rather than macrophage-specific iron deposition are likely not suitable to integrate the current data for elucidating the impact of iron on atherosclerosis.

Macrophage iron efflux is performed by ferroportin 1 (Fpn1), which is currently the only known mammalian iron exporter [19, 20]. Systemic deletion of *Fpn1* is embryonic lethal in mice, and heterozygotes with one targeted deletion of *Fpn1* appear normal [20]. A mouse strain with cell-specific deletion of *Fpn1* (*Fpn1*^*LysM/LysM*^) was generated and showed iron accumulation specifically in macrophages [21]. In this study, we generated a mouse model (*Apoe*^*−/−*^*Fpn1*^*LysM/LysM*^) by breeding *Fpn1*^*LysM/LysM*^ mice with Apolipoprotein E-deficient (*Apoe*^*−/−*^) mice, a classic mouse model of atherosclerosis, to investigate the role of macrophage iron in atherosclerosis. Here, we demonstrated that iron overload in macrophages in *Apoe*^*−/−*^*Fpn1*^*LysM/LysM*^ mice promotes foam cell formation and drastically accelerates atherosclerosis progression.

## Results

### Macrophage-specific Fpn1 deficiency drastically promotes atherosclerosis progression

To determine the role of macrophage iron accumulation in the development of atherosclerosis, we crossed *Fpn1*^*LysM/LysM*^ mice [21] with *Apoe*^*−/−*^ mice to generate *Apoe*^*−/−*^*Fpn1*^*LysM/LysM*^ mice, in which *Fpn1* was specifically deleted in macrophages on the genetic background of global *Apoe* knockout. In male *Apoe*^*−/−*^*Fpn1*^*LysM/LysM*^ and *Apoe*^*−/−*^ mice, feeding of a high fat diet was initiated at 8 weeks of age and continued for another 16 weeks to induce atherosclerosis. Hematological assessment confirmed that macrophage-specific *Fpn1* deficiency induced mild anemia (Additional file 1: Table S1 and [21]). No differences were observed in body weight or the levels of plasma lipids, including cholesterol and triglycerides, between the *Apoe*^*−/−*^*Fpn1*^*LysM/LysM*^ and *Apoe*^*−/−*^mice (Additional file 1: Table S2). More iron accumulation in plaques was confirmed in *Apoe*^*−/−*^*Fpn1*^*LysM/LysM*^ than in *Apoe*^*−/−*^mice (Additional file 1: Figure S1).

**Fig. 1:**
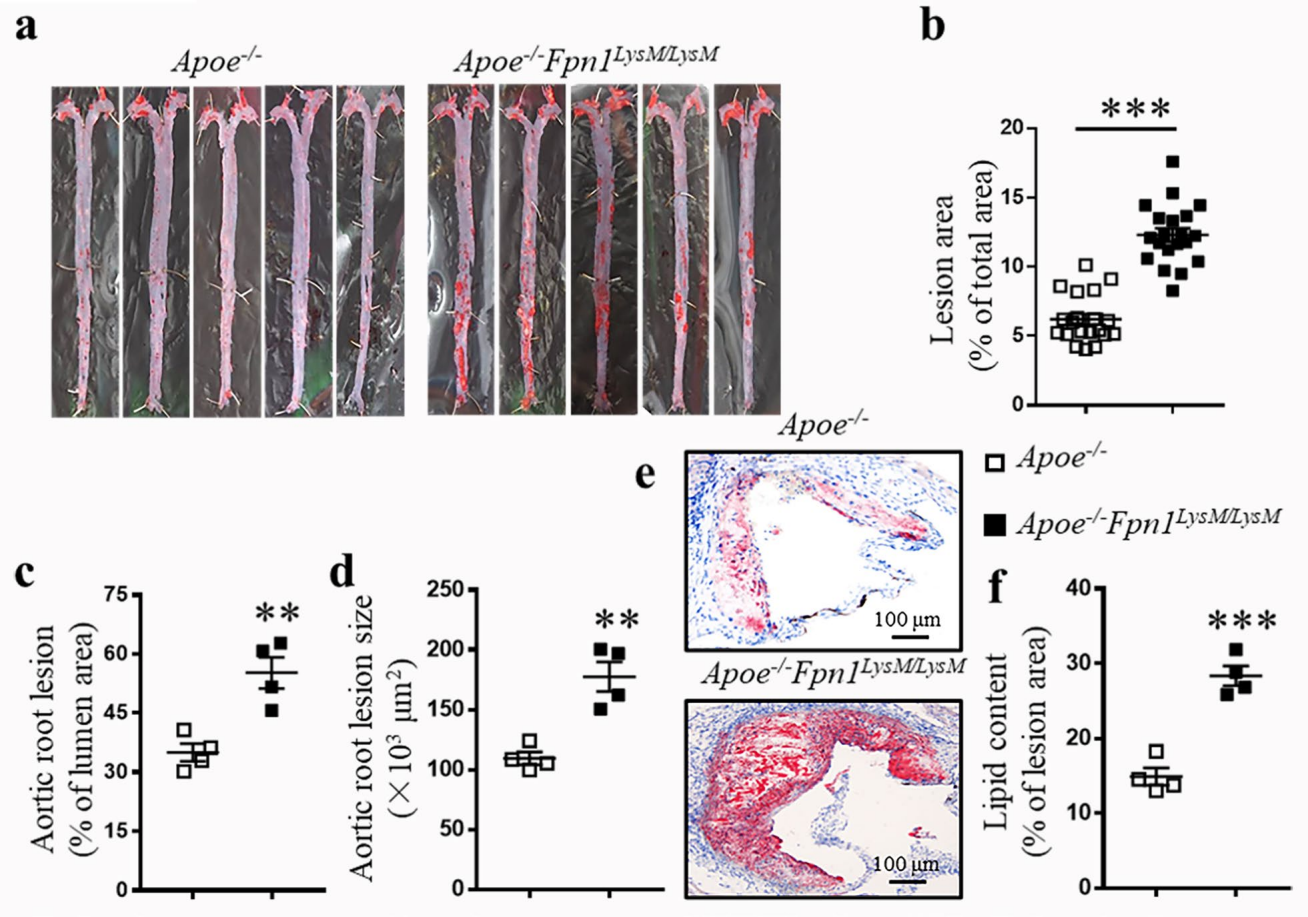
Macrophage-specific *Fpn1* deficiency promotes atherosclerosis progression. *Apoe*^*−/−*^ and *Apoe*^*−/−*^*Fpn1*^*LysM/LysM*^ male mice were fed a high fat diet for 16 weeks. **a** Representative images of Oil Red O staining of *en face* preparations of aortas.**b** Quantification of the aortic lesion area, n = 20. **c**, **d** Determination of aortic root lesion area and size, n = 4. **e** Representative images of Oil Red O staining and **f**quantification of lipid content in the aortic root in *Apoe*^*−/−*^ and *Apoe*^*−/−*^*Fpn1*^*LysM/LysM*^ mice, n = 4. Scale bar, 100 μm. Data are presented as the mean ± SEM. Statistical significance was determined using Student’s *t*-test. **P < 0.01, and ***P < 0.001 vs. *Apoe*^*−/−*^ mice

Strikingly, the severity of atherosclerosis was significantly increased after *Fpn1* depletion in mice fed with high-fat chow (Fig. 1). We quantified the plaques in *en face* preparations of the aorta. As shown in Fig. 1a, b, the percentage of lesion area in the aorta were significantly higher in *Apoe*^*−/−*^*Fpn1*^*LysM/LysM*^ mice than in *Apoe*^*−/−*^ mice, as determined by Oil Red O staining. In accordance with the increase in overall lesion area, plaque size in the aortic root was also increased in *Apoe*^*−/−*^*Fpn1*^*LysM/LysM*^ mice (Fig. 1c, d). Moreover, the Oil Red O-stained area in aortic roots presented more lipid content in *Apoe*^*−/−*^*Fpn1*^*LysM/LysM*^ mice than in *Apoe*^*−/−*^ mice (Fig. 1e, f). These results demonstrate that the *Fpn1* deletion-induced iron accumulation in plaque macrophages is associated with the severe atherosclerosis.

### Macrophage-specific Fpn1 deficiency modulates the composition of atherosclerotic plaques

The plaque composition in the aortic root was further analyzed in detail. *Apoe*^*−/−*^*Fpn1*^*LysM/LysM*^ mice exhibited significantly stronger CD68-staining intensity than their *Apoe*^*−/−*^ littermates, indicating that there were greater numbers of macrophages within the atherosclerotic lesions in the aortic roots of *Apoe*^*−/−*^*Fpn1*^*LysM/LysM*^ mice (Additional file 1: Figure S2a). Furthermore, the area of immunostaining for α-SMA and the corresponding staining intensity were also increased in *Apoe*^*−/−*^*Fpn1*^*LysM/LysM*^ mice (Additional file 1: Figure S2b), suggesting more proliferation of intimal vascular smooth muscle cells. However, plaque collagen content, as evidenced by Masson's Trichrome staining, was reduced in *Apoe*^*−/−*^*Fpn1*^*LysM/LysM*^ mice (Additional file 1: Figure S2c). In combination with the increased lesion area and plaque count, these compromised collagen levels render atherosclerotic plaques prone to rupture. Therefore, the results suggest that the atherosclerotic plaques in *Apoe*^*−/−*^*Fpn1*^*LysM/LysM*^ mice are more advanced and less stable than those in *Apoe*^*−/−*^ mice.

### Macrophage-specific Fpn1 deficiency increases oxidative stress in the aorta

**Fig. 2:**
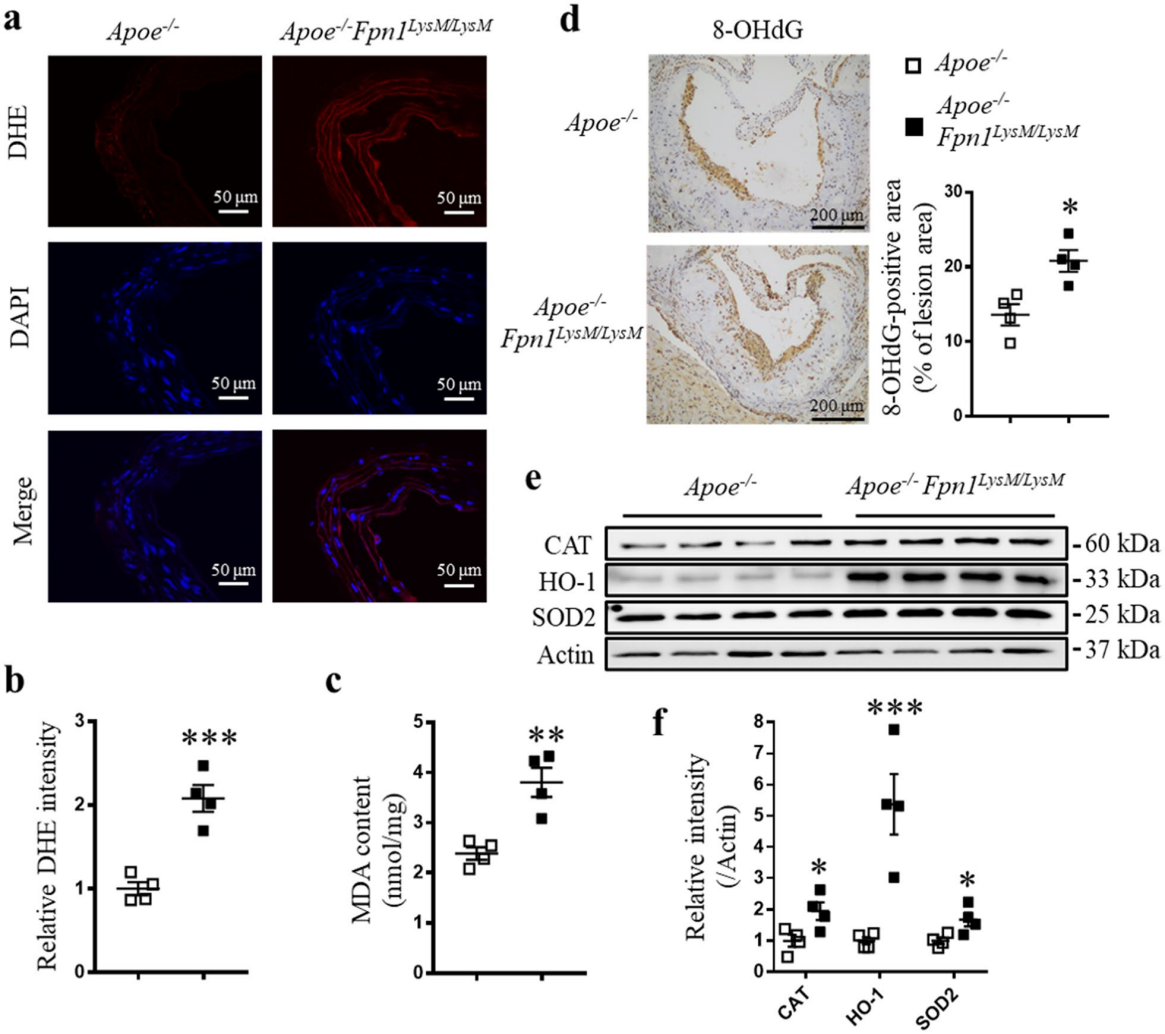
Macrophage-specific *Fpn1* deficiency increases oxidative stress in the aorta. **a** Representative images of DHE staining in the aortas of *Apoe*^*−/−*^ and *Apoe*^*−/−*^*Fpn1*^*LysM/LysM*^ mice and **b** quantification of DHE fluorescence intensity. **c** Malondialdehyde (MDA) content in the aortas of *Apoe*^*−/−*^ and *Apoe*^*−/−*^*Fpn1*^*LysM/LysM*^ mice. **d** Representative images of IHC staining for 8-OHdG and quantification of stained areas. **e** Western blot analysis of catalase (CAT), heme oxygenase 1 (HO-1) and superoxide dismutase (SOD2) protein expression in the aortas of *Apoe*^*−/−*^ and *Apoe*^*−/−*^*Fpn1*^*LysM/LysM*^ mice and **f** the quantification of this analysis. Data are presented as the mean ± SEM; n = 4. Statistical significance was determined using Student’s *t*-test. *P < 0.05, **P < 0.01, and ***P < 0.001 vs. *Apoe*^*−/−*^ mice

Iron loading may promote the formation of hydroxyl radicals via the Fenton reaction. Since oxidative stress plays a crucial role in the pathogenesis of atherosclerosis [22], we examined whether macrophage-specific *Fpn1* deficiency-mediated iron retention increased ROS levels in the aortas of *Apoe*^*−/−*^*Fpn1*^*LysM/LysM*^ mice by performing dihydroethidium (DHE) fluorescence staining and measuring malondialdehyde (MDA) content. The results revealed that the intensities of DHE and MDA were increased in *Apoe*^*−/−*^*Fpn1*^*LysM/LysM*^ mouse aortas, indicating that macrophage-specific *Fpn1* deficiency increased oxidative stress in the vascular walls (Fig. 2a–c). Oxidative damage to DNA was also assessed by immunostaining for 8-hydroxy-2′-deoxyguanosine (8-OHdG). The results showed a significant increase in the 8-OHdG-positive area in the aortic roots of *Apoe*^*−/−*^*Fpn1*^*LysM/LysM*^ mice (Fig. 2d). As a result, cellular defenses against oxidative stress should be activated. We therefore measured the protein levels of catalase (CAT), heme oxygenase 1 (HO-1), and superoxide dismutase (SOD) to assess the cellular responses to oxidative stress. Western blotting showed that the expression levels of these scavengers (CAT, HO-1, and SOD2) were all increased in the aorta (Fig. 2e, f). Among these enzymes, HO-1 presented the most remarkable change, which was correlated with increased macrophage infiltration (Additional file 1: Figure S2a). Taken together, these results suggest that iron accumulation in macrophages mediated by *Fpn1* deficiency increases oxidative stress in the aorta and promotes atherosclerosis progression.

### Macrophage-specific Fpn1 deficiency increases arterial and systemic inflammation

**Fig. 3:**
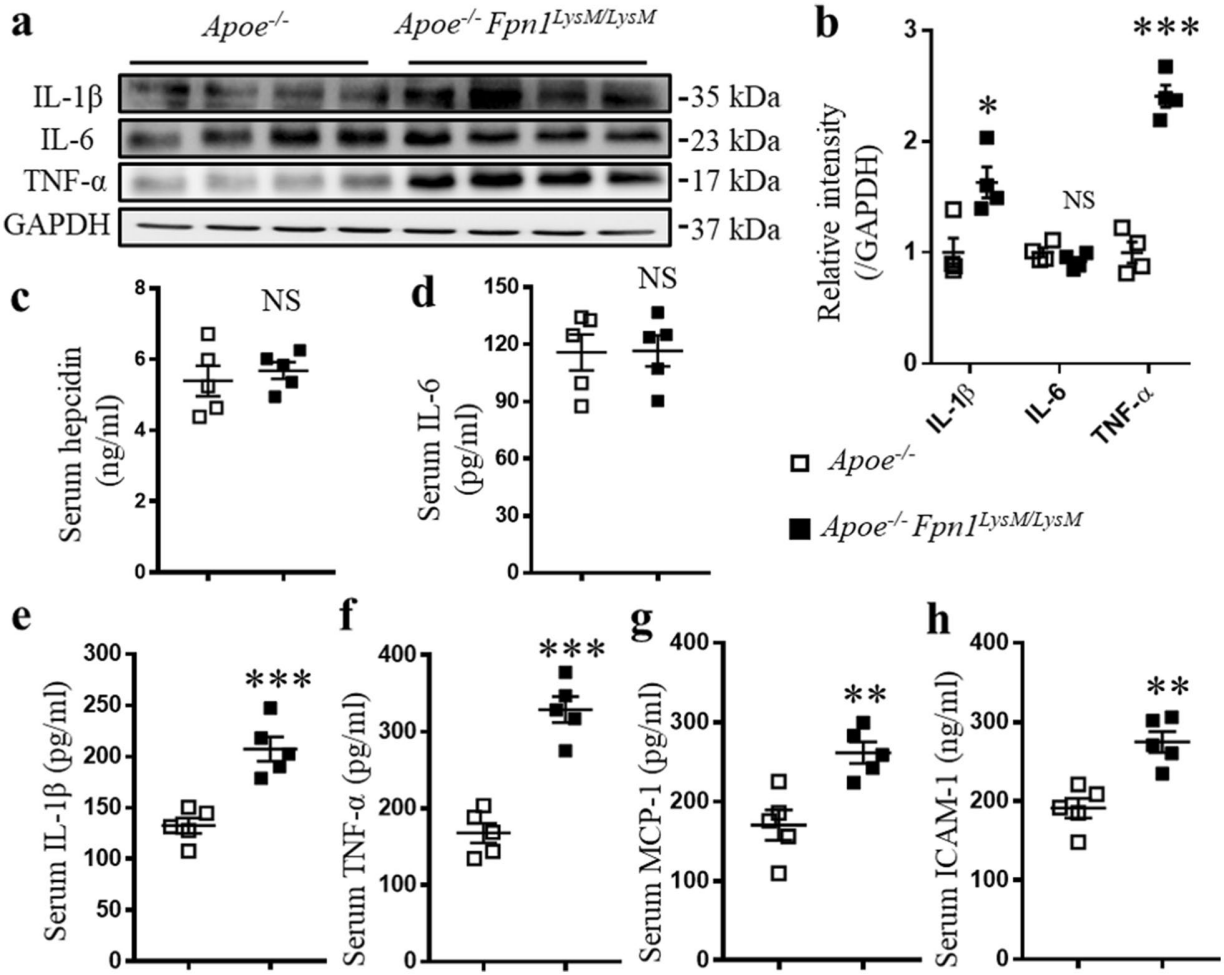
Macrophage-specific *Fpn1* deficiency increases arterial and systemic inflammation. **a**, **b** Western blot analysis of IL-1β, IL-6 and TNF-α in the aortas of *Apoe*^*−/−*^ and *Apoe*^*−/−*^*Fpn1*^*LysM/LysM*^ mice, n = 4. Determination of the concentrations of serum hepcidin (**c**), IL-6 (**d**), IL-1β (**e**), TNF-α (**f**), MCP-1 (**g**), and ICAM-1 (**h**) in *Apoe*^*−/−*^ and *Apoe*^*−/−*^*Fpn1*^*LysM/LysM*^ mice, n = 5. Data are presented as the mean ± SEM. Statistical significance was determined using Student’s *t*-test. *P < 0.05, **P < 0.01, and ***P < 0.001 vs. *Apoe*^*−/−*^ mice. NS, no significance

Macrophages play important roles in inflammatory responses, and chronic inflammation is one of the pathogenic features of atherosclerosis. Therefore, we investigated whether the loss of *Fpn1* in macrophages can enhance the secretion of cytokines during atherosclerosis progression. IL-6, IL-1β, and TNF-α are proinflammatory cytokines released by macrophages and other cell types that can produce distant inflammatory effects. Western blotting showed that the expression of IL-1β and TNF-α in the aortas of *Apoe*^*−/−*^*Fpn1*^*LysM/LysM*^ mice was significantly increased, while the protein level of IL-6 remained constant (Fig. 3a, b). The serum concentrations of hepcidin and IL-6, which were measured by ELISA, did not differ significantly between the mouse strains (Fig. 3c, d), while serum IL-1β and TNFα levels were significantly increased in *Apoe*^*−/−*^*Fpn1*^*LysM/LysM*^ mice (Fig. 3e, f). The adhesion of monocytes to the endothelium should be accelerated by monocyte chemoattractant protein (MCP)-1 and intercellular cell adhesion molecule-1 (ICAM-1). Consistent with the increased number of macrophages in plaques, the levels of MCP-1 and ICAM-1 were found to be significantly increased in the serum of *Apoe*^*−/−*^*Fpn1*^*LysM/LysM*^ mice (Fig. 3g, h). Collectively, these results suggest that *Fpn1* deficiency in macrophages increases the production of proinflammatory cytokines and promotes aortic and systemic inflammation, which is the basis of monocyte recruitment to and infiltration into plaques.

### Macrophage-specific Fpn1 deficiency accelerates foam cell formation

**Fig. 4:**
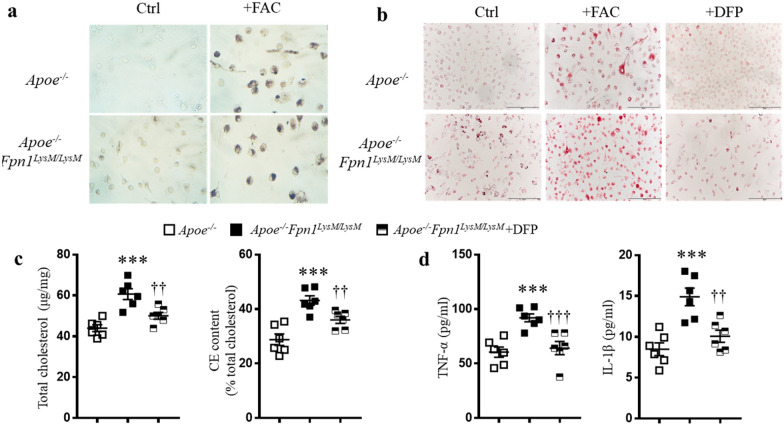
Macrophage-specific *Fpn1* deficiency exacerbates foam cell formation and proinflammatory cytokine expression. **a** Representative images of DAB-enhanced Perls’ Prussian blue staining for iron. **b** Oil red O-stained images. **c** Determination of cellular total and esterified cholesterol contents. **d** Secreted TNF-α and IL-1β levels in culture medium. Cell treatments for A–D: peritoneal cavity-derived macrophages, referred to as peritoneal macrophages, were incubated with oxLDL (50 μg/mL) in the presence or absence of DFP (50 μM) for 48 h. Data are presented as the mean ± SEM; n = 6. Statistical significance was determined using one-way ANOVA followed by Tukey’s multiple comparisons test. ***P < 0.001 *Apoe*^*−/−*^*Fpn1*^*LysM/LysM*^ vs.* Apoe*^*−/−*^, ^††^*P* < 0.01 and ^†††^*P* < 0.01* Apoe*^*−/−*^*Fpn1*^*LysM/LysM*^ + DFP vs.* Apoe*^*−/−*^*Fpn1*^*LysM/LysM*^. *FAC* ferric ammonium citrate, *DFP* deferiprone, *oxLDL* oxidized low-density lipoprotein

The formation of foam cells from macrophages is a crucial step in the development of atherosclerosis. To determine whether macrophage-specific *Fpn1* deficiency-induced iron retention affected foam cell formation, we isolated primary peritoneal macrophages from mice and loaded the cells with oxLDL (50 μg/ml) for 48 h in the presence or absence of 100 μM FAC, an iron source, or 50 μM DFP, an iron chelator. DAB-enhanced Perls’ Prussian blue staining showed extensive iron accumulation in *Apoe*^*−/−*^*Fpn1*^*LysM/LysM*^ macrophages, and this staining was further enhanced by treatment with FAC, confirming that the staining was iron specific and that *Fpn1* deficiency led to intracellular iron accumulation (Fig. 4a). After treatment with oxLDL, Oil Red O staining was performed. The results showed that more lipids accumulated in the *Apoe*^*−/−*^*Fpn1*^*LysM/LysM*^ macrophages than in *Apoe*^*−/−*^ cells, and this accumulation was significantly enhanced by treatment with FAC and reduced by treatment with DFP, indicating that iron overload strengthened lipid deposition (Fig. 4b). Consistent with the Oil Red O staining results, the levels of total and esterified cholesterol were significantly increased in *Apoe*^*−/−*^*Fpn1*^*LysM/LysM*^ macrophages compared with *Apoe*^*−/−*^ macrophages, whereas iron chelation reduced cholesterol levels (Fig. 4c). Next, we measured the cytokines released by the macrophages. Culture medium from *Apoe*^*−/−*^*Fpn1*^*LysM/LysM*^ macrophages exhibited higher levels of both TNF-α and IL-1β than medium from *Apoe*^*−/−*^ macrophages, while iron chelation suppressed the release of these proinflammatory factors (Fig. 4d). These results indicate that *Fpn1* deficiency-mediated iron accumulation dramatically increases the potential of macrophages to form foam cells.

### Fpn1 deficiency-mediated iron accumulation in macrophages suppresses ABC transporters through downregulated LXRα expression

**Fig. 5:**
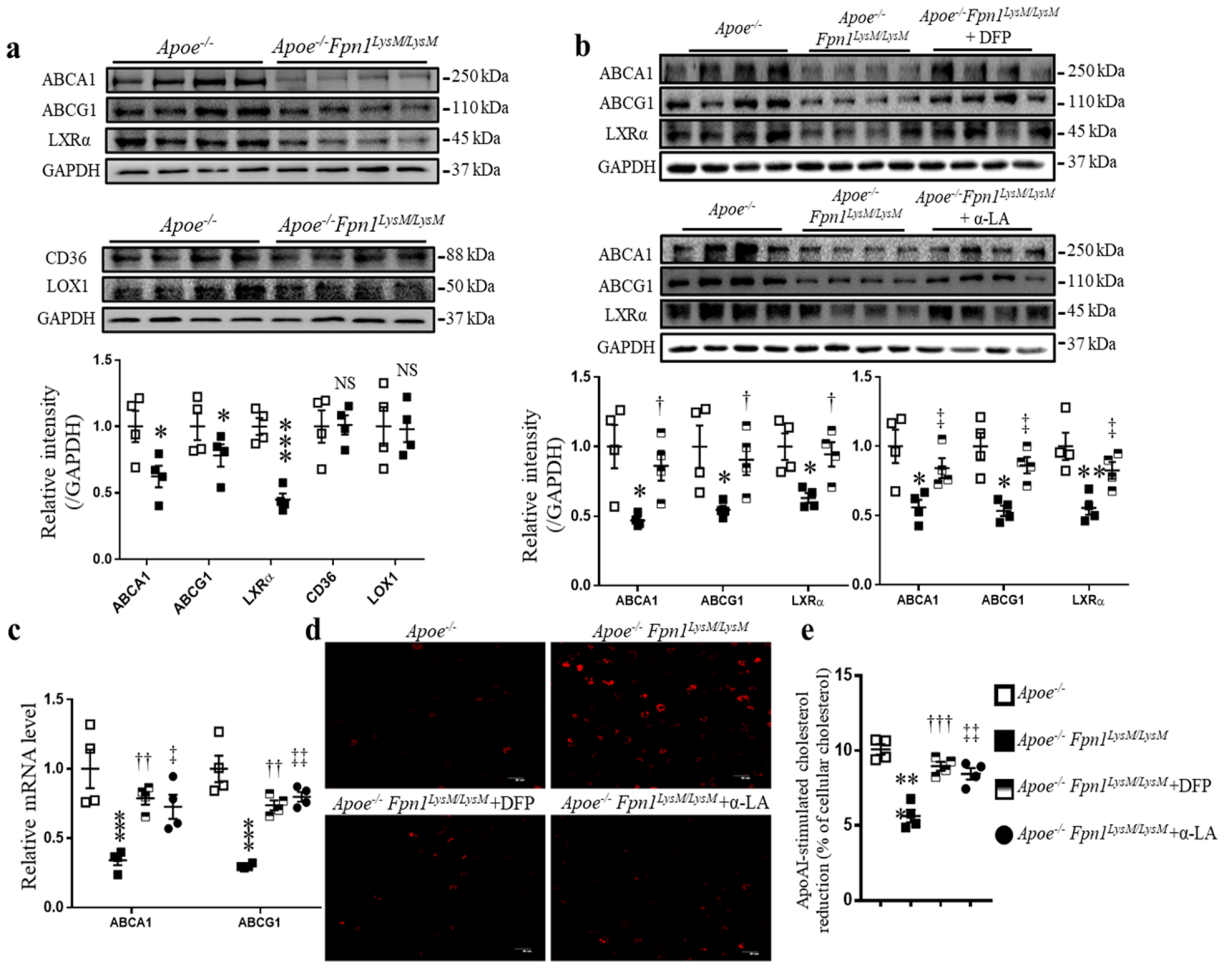
Macrophage-specific *Fpn1* deficiency suppresses ABC transporters by downregulating LXRα expression. Peritoneal macrophages from *Apoe*^*−/−*^ and *Apoe*^*−/−*^*Fpn1*^*LysM/LysM*^ mice were collected, cultured, and treated with the iron chelator DFP or the antioxidant α-LA as indicated. **a** Protein levels of ABCA1, ABCG1, LXRα, CD36 and LOX1 revealed by Western blot analysis. **b** Protein levels of ABCA1, ABCG1, and LXRα after the macrophages were treated with DFP (50 μM) or α-LA (200 nM) for 48 h. **c** The relative mRNA levels of ABCA1 and ABCG1, as determined by qPCR. **d** Representative images of DHE staining following treatment with DFP (50 μM) or α-LA (200 nM) for 48 h. **e** ApoAI-mediated cholesterol efflux in *Apoe*^*−/−*^*Fpn1*^*LysM/LysM*^ macrophages. The cholesterol-loaded macrophages were incubated with or without ApoAI (100 µg/ml) in the presence or absence of DFP (50 μM) or α-LA (200 nM) for 24 h. The results are expressed as the percentage change in the intracellular total cholesterol amount in the presence of ApoAI relative to that in ApoAI-free medium. Data are presented as the mean ± SEM; n = 4. Statistical significance was determined using Student’s *t*-test and one-way ANOVA followed by Tukey’s multiple comparisons test. *P < 0.05, **P < 0.01, and ***P < 0.001* Apoe*^*−/−*^*Fpn1*^*LysM/LysM*^ vs.* Apoe*^*−/−*^; ^†^*P* < 0.05, ^††^*P* < 0.01 and ^†††^*P* < 0.001* Apoe*^*−/−*^*Fpn1*^*LysM/LysM*^ + DFP vs.* Apoe*^*−/−*^*Fpn1*^*LysM/LysM*^; ^‡^*P* < 0.05, ^‡‡^*P* < 0.01* Apoe*^*−/−*^*Fpn1*^*LysM/LysM*^ + α-LA vs.* Apoe*^*−/−*^*Fpn1*^*LysM/LysM*^. *α-LA* alpha lipoic acid

Both uncontrolled uptake of modified LDL and impaired cholesterol efflux lead to lipid accumulation. Therefore, we asked whether the observed iron overload could cause uncontrolled uptake of modified LDL or impaired cholesterol efflux in *Fpn1*-deficient macrophages. To this end, we examined the expression of ABCA1 and ABCG1, two important transporters mediating cholesterol efflux, and CD36 and LOX1, two receptors responsible for the uptake of oxLDL. *Apoe*^*−/−*^*Fpn1*^*LysM/LysM*^ macrophages expressed significantly lower levels of ABCA1 and ABCG1 than *Apoe*^*−/−*^ cells, while no difference in the expression of CD36 or LOX1 was found between *Apoe*^*−/−*^ and *Apoe*^*−/−*^*Fpn1*^*LysM/LysM*^ macrophages (Fig. 5a), suggesting that compromised efflux of cholesterol occurred when iron was overloaded in the macrophages.

Liver X receptors (LXRs) are transcriptional regulators of lipid homeostasis that play an important role in the development of atherosclerosis (see review [23]). We thus asked whether LXRα expression was downregulated to reduce the expression of ABCA1 and ABCG1 in *Fpn1*-depleted macrophages. As expected, the protein level of LXRα was downregulated in *Fpn1*-depleted macrophages (Fig. 5a). To further examine whether the high level of intracellular iron, mediated by *Fpn1* depletion, caused the change in LXRα expression, we treated *Apoe*^*−/−*^*Fpn1*^*LysM/LysM*^ macrophages with DFP to reduce their iron content. Treatment with DFP significantly increased the expression of LXRα, as revealed by Western blot analysis (Fig. 5b). In addition, the protein and mRNA levels of ABCA1/ABCG1 were also increased by treatment with DFP (Fig. 5b, c). These data suggest that iron overload represses LXRα expression and subsequently reduces the expression of the cholesterol exporters ABCA1 and ABCG1 to enhance cellular lipid retention and promote macrophage differentiation into foam cells.

In *Apoe*^*−/−*^*Fpn1*^*LysM/LysM*^ mice, iron overload increased oxidative stress, which could play critical roles in the regulation of various cell functions and biological processes. Here, we postulate that ROS are the critical factors in foam cell formation, more precisely in the downregulation of lipid exporters; thus, we chose an antioxidant, α-LA, to modulate the differentiation of macrophages. We first used DHE fluorescence staining to evaluate ROS production. *Apoe*^*−/−*^*Fpn1*^*LysM/LysM*^ macrophages presented a higher intensity of red fluorescence than *Apoe*^*−/−*^ macrophages, and both DFP and α-LA treatment reduced the ROS levels (Fig. 5d). Interestingly, Western blot and qPCR analyses showed that treatment with α-LA significantly increased the expression of ABCA1/ABCG1 at both the protein and mRNA levels and that their upstream transcription factor subunit LXRα was also upregulated after treatment with α-LA (Fig. 5b, c), supporting the idea that iron-induced oxidative stress played an important role in blocking lipid efflux via LXRα repression [24].

Since *Fpn1 *deficiency decreases the expression of ABCA1 and ABCG1 in macrophages, in vitro functional assays were performed to detect the capacity for cholesterol efflux mediated by ApoAI, a major structural protein of high-density lipoprotein involved in cellular cholesterol efflux. We incubated macrophages with oxLDL (50 μg/ml) for 48 h to induce cholesterol accumulation and then exposed the cells to ApoAI (100 μg/ml) for 24 h to induce cholesterol efflux in the presence or absence of DFP or α-LA. Intracellular total cholesterol levels were determined by enzymatic assays. The results showed that ApoAI-stimulated cholesterol efflux in *Apoe*^*−/−*^*Fpn1*^*LysM/LysM*^ macrophages was markedly weaker than that in *Apoe*^*−/−*^, *Apoe*^*−/−*^*Fpn1*^*LysM/LysM*^ + DFP and *Apoe*^*−/−*^*Fpn1*^*LysM/LysM*^ + α-LA macrophages (Fig. 5e).

### ***Iron chelation therapy prevents severe atherosclerosis in Apoe***^***−/−***^***Fpn1***^***LysM/LysM***^*** mice***

**Fig. 6:**
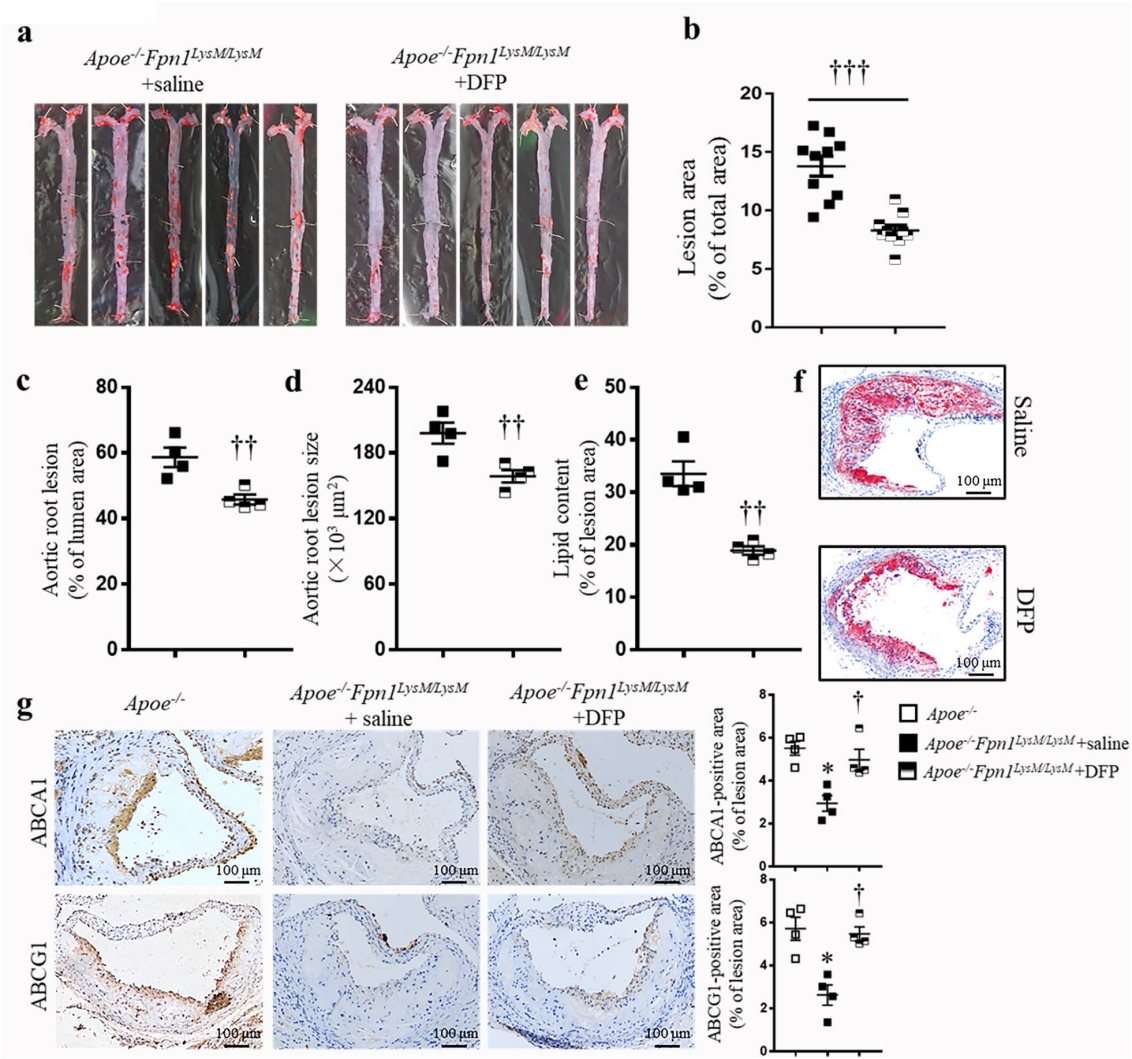
Iron chelation therapy prevents severe atherosclerosis in *Apoe*^*−/−*^* Fpn1*^*LysM/LysM*^ mice. Eight-week-old male *Apoe*^*−/−*^*Fpn1*^*LysM/LysM*^ mice were fed a High fat diet and randomly divided into 2 groups: the vehicle—(saline) and iron chelator-treated (DFP, 80 mg/kg) groups. **a** Representative images of Oil Red O staining of aortas. **b** Quantification of the aortic lesion area, n = 10. **c**, **d** Determination of aortic root lesion area and size, n = 4. **e**, **f** Representative images and quantification of Oil Red O staining of the aortic roots of *Apoe*^*−/−*^*Fpn1*^*LysM/LysM*^ mice, n = 4. **g** Representative images of IHC staining for ABCA1 and ABCG1 and the quantification of the stained areas, n = 4. Scale bar, 100 μm. Data are presented as the mean ± SEM. Statistical significance was determined using Student’s *t*-test and one-way ANOVA followed by Tukey’s multiple comparisons test. *P < *0.05 Apoe*^*−/−*^*Fpn1*^*LysM/LysM*^ + saline vs.* Apoe*^*−/−*^ mice, ^†^*P* < 0.05, ^†††^*P* < 0.001* Apoe*^*−/−*^*Fpn1*^*LysM/LysM*^ + DFP *vs. Apoe*^*−/−*^*Fpn1*^*LysM/LysM*^ + saline

Since treatment with the iron chelator DFP reduced intracellular iron levels and lipid deposits ex vivo, we hypothesized that DFP would reverse lipid accumulation and diminish plaque formation in vivo. Therefore, we administered DFP to 8-week-old *Apoe*^*−/−*^*Fpn1*^*LysM/LysM*^ mice maintained on a high fat diet for 16 weeks. Hematological assessment showed that red blood cell counts, hemoglobin levels, hematocrit values and mean corpuscular hemoglobin values were reduced in the iron chelation group (DFP) compared with the vehicle group (Additional file 1: Table S3). The serum iron level and transferrin saturation were significantly lower in the DFP-treated mice (Additional file 1: Table S3). No differences were observed in body weight or the levels of plasma lipids, including cholesterol and triglycerides, between the DFP-treated and saline-treated mice (Additional file 1: Table S4). However, DFP administration significantly reduced lesion area, as revealed by *en face* preparations of the aorta for Oil Red O staining (Fig. 6a, b). In agreement with these observations, plaque size in the aortic root decreased after DFP administration, as indicated by the measured reductions in lesion percentage and lesion size (Fig. 6c, d). Moreover, the Oil Red O-stained area in the aortic root also showed less lipid content in the DFP-treated mice than in the saline-treated mice (Fig. 6e, f). In addition, the expression of ABCA1/ABCG1 was increased within plaques after DFP administration (Fig. 6g). These data demonstrated that chronic systemic iron chelation has therapeutic effect in *Apoe*^*−/−*^*Fpn1*^*LysM/LysM*^ mouse model of atherosclerosis.

## Discussion

We report here that *Fpn1* deficiency in macrophages dramatically accelerates the progression of atherosclerosis in mice despite the mice with mild anemia and without significant change of plasma hepcidin levels. These results provide direct evidence for the local contribution of macrophage iron to atherosclerosis development. Moreover, we report that iron accumulation mediated by *Fpn1* deficiency in macrophages promotes lipid retention for foam cell formation via downregulated LXRα expression (model in Additional file 1: Figure S3).

Although iron accumulates in human atherosclerotic lesions [25], epidemiological and experimental studies have produced controversial results regarding the role of iron in atherosclerosis development and progression. Considering the natural function of macrophages in iron recycling, macrophages need to export iron by, at least partially, the currently only known transporter Fpn1. In the classical HH patients and animal models, Fpn1 function is enhanced by downregulated hepcidin [16, 26], which may explain systemic iron overload in HH patients. Though, systemic iron overload is not a risk factor in atherosclerotic patients [7, 8]. Later, the role of macrophage iron was proposed. Given these findings and the proposal, we generated *Apoe*^*−/−*^*Fpn1*^*LysM/LysM*^ mice to investigate the role of macrophage iron. The symptoms of the mice presented a severe atherosclerosis as observed clinically. A very recent report has demonstrated that hepcidin deficiency protects against atherosclerosis in a hyperlipidemic mouse (*Hamp*^*−/−*^*/Ldlr*^*−/−*^) model, in which hepcidin deficiency is associated with both an increased serum iron level and a decreased macrophage iron level [16]. The data from both *Hamp*^*−/−*^*/Ldlr*^*−/−*^ [16] and *Apoe*^*−/−*^*Fpn1*^*LysM/LysM*^ (this work) models reciprocally demonstrated that low levels of iron in macrophages induced by upregulated Fpn1 protected against atherosclerosis and that high levels of iron in macrophages induced by *Fpn1* depletion accelerated the progression of atherosclerosis. These results were further confirmed by the observation in this study that *Apoe*^*−/−*^*Fpn1*^*LysM/LysM*^ mice developed typical atherosclerotic plaques at 6 months of age more severely than *Apoe*^−/−^ mice when fed a normal diet (results not shown). Thus, we emphasize the important role of macrophage iron that differs from the role of systemic iron overload in HH patients because macrophages in classical HH patients do not overload iron.

Very interestingly, another mouse model (*Apoe*^*−/−*^*Fpn*^wt/C326S^), generated by Vinchi recently [17], presented aggravated atherosclerosis. This *Fpn* mutation (C326S) leads to type IV HH by rendering Fpn1 resistant to hepcidin binding, internalization, and degradation [27]. Another mouse model with mutation (*ffe*, H32R) of Fpn1 combining *Apoe*^*−/−*^ to generate mice with macrophage-specific iron accumulation [18]. Both mutations are dominant negative and animals homozygous for the mutations die early in gestation. The former mutation produced the aggravated atherosclerosis, compared with the control mice, as we observed in this study. However, the mice *Apoe*^−/−^*/ffe* did not exhibit more severe atherosclerosis than *Apoe*^−/−^ mice. Comparing with *Apoe*^−/−^*Fpn *^*LysM/LysM*^ mice, *Apoe*^−/−^*Fpn1*^wt/C326S^ and *Apoe*^−/−^*ffe* mice may have less iron accumulation in macrophages, theoretically, due to a small portion of functional Fpn1 in the two heterozygous mutants though its gain-of function mutation. And the effects of mutation C326S might be stronger than H32R mutation, which is supported by patients with C326S mutation and none with H32R (H30 in human) so far and by remarkably diminished binding capacity of C326S mutant with hepcidin [27]. Taken together, the content of macrophage iron matters when the deposited iron reaches a certain level and acts as a detrimental factor to aggravate atherosclerosis. Clinically, the deposited iron might not result from deficiency of functional Fpn1. We found significantly low levels of the ferroxidases, ceruloplasmin and hephaestin, in plaques comparing to normal vessel tissue, whereas the levels of ferritin and FPN1 were quite high [4]. The accumulated iron may result from the inability to be released due to the insufficient ferroxidase. Thus, systemic iron chelation would be beneficial, as shown in our study and others (reviewed in [28]).

Due to the significant role of accumulated iron in generating ROS via the Fenton reaction, one important aspect in *Apoe*^*−/−*^*Fpn1*^*LysM/LysM*^ mice was oxidative stress, which was significantly higher than in *Apoe*^*−/−*^ mice. It has been shown that ROS stress promotes foam cell formation. Here, we provide more evidence to support the role of iron-dependent oxidative stress in suppressing cholesterol efflux through the downregulation of ABC transporter expression. It is well accepted that LXRα and LXRβ complexes control cholesterol removal from macrophages by upregulating the expression of ABC transporters, including ABCA1 and ABCG1 [29]. We further demonstrate that the suppression of LXRα decreases the expression of ABCA1 and ABCG1 as occurred in Kupffer cells (reviewed in [30]). Notably, Bories et al. demonstrated that iron loading in IL-4-polarized M2 macrophages drove the activation of LXRα and enhanced the transcription of ABCA1/ABCG1 [31]. Although discussing each previously published result that contradicts our findings is beyond the scope of this manuscript, one major distinction that deserves attention is the different approaches that have been used to alter macrophage plasticity. In our study, the plasticity of *Fpn1*-deleted macrophages was directly shaped by retained iron, whereas the plasticity of IL-4-induced M2-like macrophages was directed by iron-induced ferroportin expression [28]. Therefore, the macrophage phenotypes are distinct with more M1-like macrophages in our study and more M2-like macrophages in Bories’ study. As reviewed in [24], M2-like macrophages undergo a protective response to erythrophagocytosis and to oxidized LDL. It also supports that hemoglobin-stimulated macrophages display reduced intracellular iron content through upregulation of ferroportin expression, which in turn reduces iron-induced oxidative stress and further increases the expression of ABCA1/ABCG1 through the activation of LXRα [32].

## Conclusions

In summary, the present study provided direct evidence that iron accumulation in macrophages accelerated the development of atherosclerosis. The interaction of iron and lipid metabolism takes place in macrophage-rich atherosclerotic plaques. And we also suggest that altering intracellular iron levels in macrophages by systemic iron chelation or dietary iron restriction may be a potential supplementary strategy to limit or even regress the progression of atherosclerosis.

## Materials and methods

Detailed information on the methods used for histological, biochemical, and enzymatic assays is available in the supplementary data.

### Animals

*Apoe*^*−/−*^ mice were obtained from the Model Animal Research Center of Nanjing University (Nanjing, China). *Fpn1*-floxed (*Fpn1*^*flox/flox*^) mice on the 129/SvEvTac background were generated in our previous study [21]. *Fpn1*^*flox/flox*^ mice were mated with *LysM-Cre* mice to create *LysM*(*Cre/Cre*) *Fpn1*(*flox/flox*) (named *Fpn1*^*LysM/LysM*^) mice in which *Fpn1* was specifically deleted in macrophages. *Fpn1*^*LysM/LysM*^ and *Fpn1*^*flox/flox*^ mice were crossed with *Apoe*^*−/−*^ mice (C57BL/6J background) and backcrossed for more than 10 generations to generate *Apoe*^*−/−*^*Fpn1*^*LysM/LysM*^ and *Apoe*^*−/−*^* Fpn1*^*flox/flox*^ (named *Apoe*^*−/−*^) mice before any experimentation was performed. At the age of 8 weeks, male *Apoe*^*−/−*^ mice and their *Apoe*^*−/−*^*Fpn1*^*LysM/LysM*^ littermates were fed a high fat diet (0.2% cholesterol and 20% fat) for 16 weeks in a SPF animal facility with a normal 12-h light-and-dark cycle and controlled temperature conditions (25 °C). All animals were anesthetized with an intraperitoneal injection of pentobarbital sodium (40 mg/kg) and euthanized by cervical dislocation. For sample collection, all protocols were approved by the Animal Investigation Ethics Committee of The Affiliated Drum Tower Hospital of Nanjing University Medical School and were performed according to the Guidelines for the Care and Use of Laboratory Animals published by the National Institutes of Health, USA.

### Treatment with an iron chelator

*Apoe*^*−/−*^*Fpn1*^*LysM/LysM*^ mice were randomly divided into 2 groups: the vehicle (saline injection) and iron chelation (treatment with deferiprone, DFP, Sigma-Aldrich, St. Louis, MO) groups. DFP at a dose of 80 mg/kg or the same-volume saline was administered to these 8-week-old mice (fed a high fat diet) by daily intraperitoneal injection for 16 weeks. No mice were excluded during the experiments.

### Cell culture

Peritoneal macrophages were collected from peritoneal exudates 4 days after injecting 8-week-old mice with 0.3 ml of 4% BBL thioglycollate, Brewer modified (BD Biosciences, Shanghai, China), and then cultured in RPMI 1640 medium supplemented with 10% fetal bovine serum (FBS) for 8 h. The macrophages were then cultured in medium containing 50 μg/ml human oxidized low-density lipoprotein (oxLDL) for 48 h in the presence of 100 μM ferric ammonium citrate (FAC), 50 μM DFP or 200 nM alpha lipoic acid (α-LA; an antioxidant). Oil Red O staining was performed to evaluate foam cell formation. Cellular iron staining was performed using Perls’ Prussian blue stain. The protein levels of ABCA1, ABCG1, CD36, LOX-1 and LXRα were determined by Western blot analysis.

### Statistical analysis

All experiments were randomized and blinded. All the data are presented as the mean ± SEM. A two-tailed Student’s *t*-test (for two groups) or one-way analysis of variance followed by multiple comparisons test with Bonferroni correction (for more than two groups) was performed by using SPSS 17.0 (SPSS Inc, Chicago, IL). *P* < 0.05 indicated statistical significance.

## Supplementary information

**Additional file 1: Figure S1.** Macrophage-specific *Fpn1* deletion causes iron accumulation in plaques. (A) Western blot analysis of ferritin protein in aortas of *Apoe*^*−/−*^ and *Apoe*^*−/−*^*Fpn1*^*LysM/LysM*^ mice. (B) Iron content of aorta determined by using colorimetric ferrozine-based assays. n = 4. (C) DAB-enhanced Perls’ stain for iron in plaques. The results are shown as the mean ± SEM. Statistical significance was determined using Student’s t-test. **P < 0.01 vs. *Apoe*^−/−^ mice. **Figure S2.** Macrophage-specific *Fpn1* deficiency modulates the composition of atherosclerotic plaques. Representative images and quantification of IHC staining for CD68 (a) and α-SMA (b) and Masson trichrome staining for collagenous fibers (c) in the aortic roots of *Apoe*^*−/−*^ and *Apoe*^*−/−*^*Fpn1*^*LysM/LysM*^ mice. Scale bar, 100 μm. The quantification of stained areas is presented as the mean ± SEM; n = 4. Statistical significance was determined using Student’s *t*-test. **P < 0.01, and ***P < 0.001 vs. *Apoe*^*−/−*^ mice. **Figure S3.** Schematic model for the effects of accumulated iron in macrophages on foam cell formation. Macrophage iron retention (induced here by *Fpn1* deletion) triggers oxidative stress, which inhibits LXRα-mediated transcription of ABCA1/ABCG1 to suppress cholesterol efflux. This program promotes foam cell formation and further atherosclerosis development. **Table S1.** Serum and hematologic parameters of *Apoe*^*−/−*^ and *Apoe*^*−/−*^*Fpn1*^*LysM/LysM*^ mice. **Table S2.** Body weight and plasma lipids of *Apoe*^*−/−*^ and *Apoe*^*−/−*^*Fpn1*^*LysM/LysM*^ mice. **Table S3.** Serum and hematologic parameters of *Apoe*^*−/−*^*Fpn1*^*LysM/LysM*^ mice injected with saline or DFP. **Table S4.** Body weight and plasma lipids of *Apoe*^*−/−*^*Fpn1*^*LysM/LysM*^ mice injected with saline or DFP. **Additional Methods**.

## Data Availability

The data generated or analyzed during this study are included in this published article and its additional information files.

## Abbreviations

CAD: Coronary artery disease
ROS: Reactive oxygen species
HH: Hereditary hemochromatosis
Fpn1: Ferroportin 1
*Apoe*^*−*^^*/*^^*−*^: Apolipoprotein E-deficient
DFP: Deferiprone
oxLDL: Oxidized low-density lipoprotein
FAC: Ferric ammonium citrate
α-LA: Alpha lipoic acid
DHE: Dihydroethidium
MDA: Malondialdehyde
8-OHdG: 8-Hydroxy-2′-deoxyguanosine
CAT: Catalase
HO-1: Heme oxygenase 1
SOD: Superoxide dismutase
MCP-1: Monocyte chemoattractant protein-1
ICAM-1: Intercellular cell adhesion molecule-1
LXRs: Liver X receptors
