# Supplementary material for: Iron accumulation in macrophages promotes the formation of foam cells and development of atherosclerosis
Source: Cell Biosci. 2020 Nov 26;10:137. doi: 10.1186/s13578-020-00500-5 (PMC7691057; doi:10.1186/s13578-020-00500-5)
Supplement: Supplementary file 1 — Additional file 1: Figure S1. Macrophage-specific Fpn1 deletion causes iron accumulation in plaques. (A) Western blot analysis of ferritin protein in aortas of Apoe−/− and Apoe−/−Fpn1LysM/LysM mice. (B) Iron content of aorta determined by using colorimetric ferrozine-based assays. n = 4. (C) DAB-enhanced Perls’ stain for iron in plaques. The results are shown as the mean ± SEM. Statistical significance was determined using Student’s t-test. **P < 0.01 vs. Apoe−/− mice. Figure S2. Macrophage-specific Fpn1 deficiency modulates the composition of atherosclerotic plaques. Representative images and quantification of IHC staining for CD68 (a) and α-SMA (b) and Masson trichrome staining for collagenous fibers (c) in the aortic roots of Apoe−/− and Apoe−/−Fpn1LysM/LysM mice. Scale bar, 100 μm. The quantification of stained areas is presented as the mean ± SEM; n = 4. Statistical significance was determined using Student’s t-test. **P < 0.01, and ***P < 0.001 vs. Apoe−/− mice. Figure S3. Schematic model for the effects of accumulated iron in macrophages on foam cell formation. Macrophage iron retention (induced here by Fpn1 deletion) triggers oxidative stress, which inhibits LXRα-mediated transcription of ABCA1/ABCG1 to suppress cholesterol efflux. This program promotes foam cell formation and further atherosclerosis development. Table S1. Serum and hematologic parameters of Apoe−/− and Apoe−/−Fpn1LysM/LysM mice. Table S2. Body weight and plasma lipids of Apoe−/− and Apoe−/−Fpn1LysM/LysM mice. Table S3. Serum and hematologic parameters of Apoe−/−Fpn1LysM/LysM mice injected with saline or DFP. Table S4. Body weight and plasma lipids of Apoe−/−Fpn1LysM/LysM mice injected with saline or DFP. Additional Methods. [file 13578_2020_500_MOESM1_ESM.docx]

**Additional figures**


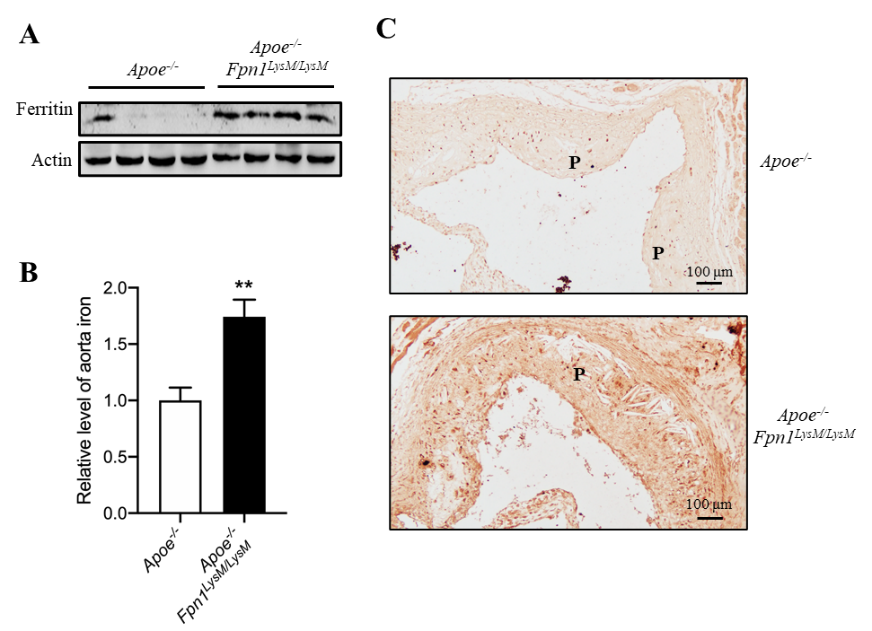


**Figure S1. Macrophage-specific *Fpn1* deletion causes iron accumulation in plaques**. (A) Western blot analysis of ferritin protein in aortas of *Apoe^-/-^* and *Apoe^-/-^Fpn1^LysM/LysM^* mice. (B) Iron content of aorta determined by using colorimetric ferrozine-based assays. n = 4. (C) DAB-enhanced Perls’ stain for iron in plaques. The results are shown as the mean ± SEM. Statistical significance was determined using Student’s t-test. **P < 0.01 vs. *Apoe*^-/-^ mice.


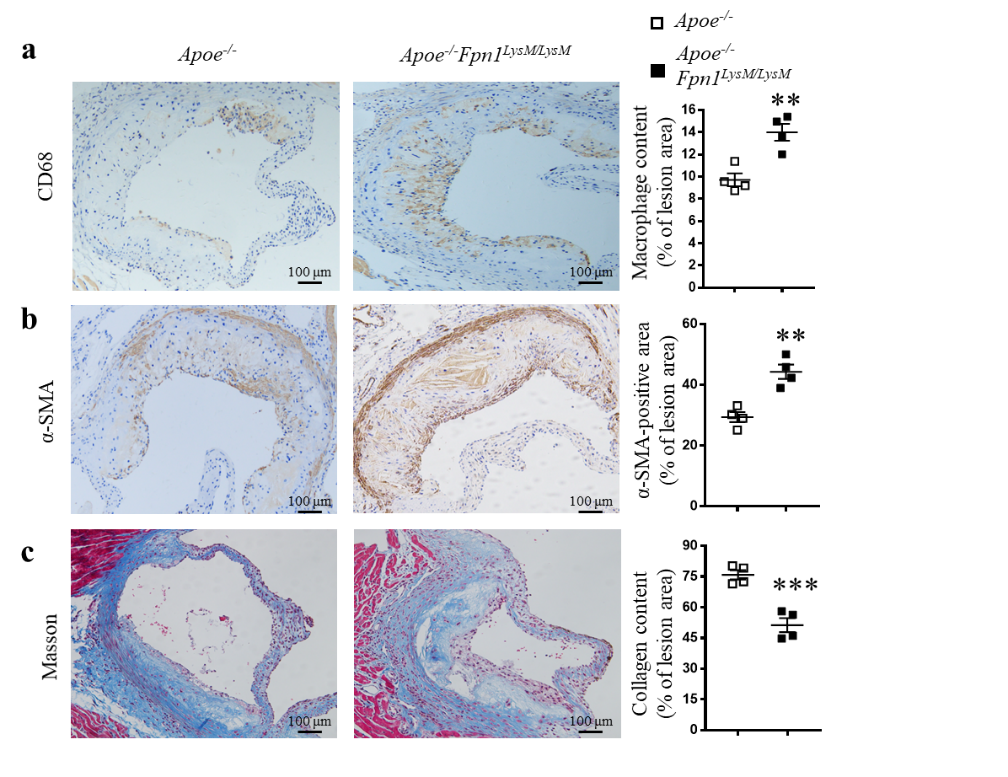


**Figure S2. Macrophage-specific *Fpn1* deficiency modulates the composition of atherosclerotic plaques.** Representative images and quantification of IHC staining for CD68 (a) and α-SMA (b) and Masson trichrome staining for collagenous fibers (c) in the aortic roots of *Apoe^-/-^* and *Apoe^-/-^Fpn1^LysM/LysM^* mice. Scale bar, 100 μm. The quantification of stained areas is presented as the mean ± SEM; n = 4. Statistical significance was determined using Student’s *t*-test. ***P* < 0.01, and ****P < 0.001* *vs*. *Apoe^-/-^* mice.


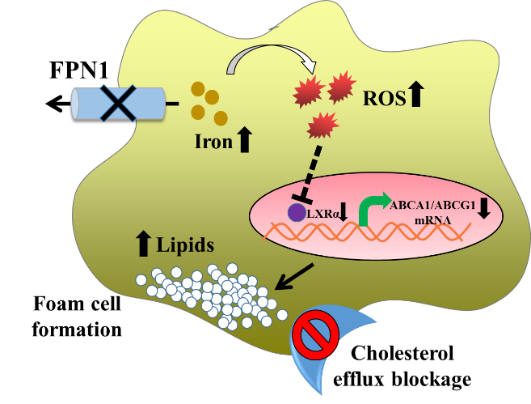


**Figure S3. Schematic model for the effects of accumulated iron in macrophages on foam cell formation**. Macrophage iron retention (induced here by *Fpn1* deletion) triggers oxidative stress, which inhibits LXRα-mediated transcription of ABCA1/ABCG1 to suppress cholesterol efflux. This program promotes foam cell formation and further atherosclerosis development.

**Additional tables**

**Table S1 Serum and hematologic parameters of *Apoe^-/-^* and *Apoe^-/-^Fpn1^LysM/LysM^* mice.**

|  | ***Apoe^-/-^*** | ***Apoe^-/-^Fpn1^LysM/LysM^*** | ***P*** |
| --- | --- | --- | --- |
| **Serum parameters** | | | |
| Serum iron, μg/dL | 124±12 | 114±15 | < 0.05 |
| UIBC, μg/dL | 189±25 | 195±30 | > 0.05 |
| TIBC, μg/dL | 311±33 | 309±39 | > 0.05 |
| TS, % | 40.1±2.9 | 37.0±3.4 | < 0.01 |
| **Hematology** | | | |
| WBCs (x10^9^/L) | 4.3±1.3 | 5.1±2.3 | > 0.05 |
| RBCs (x10^12^/L) | 9.4±0.3 | 9.0±0.7 | > 0.05 |
| Hb (g/L) | 136±8 | 118±13 | < 0.05 |
| Hct (%) | 42.1±1.8 | 35.5±3.7 | < 0.001 |
| MCV (fl) | 44.8±1.0 | 39.5±1.3 | < 0.001 |
| MCH (pg) | 14.4±0.4 | 13.1±1.1 | < 0.001 |

Eight-weeks old mice were fed the Western diet for another 16 weeks. Blood was drawn (detail sees ***Blood samples and tissue collection***). Data are represented as mean ± SEM. Statistical significance was determined using Student’s *t*-test. UIBC, unsaturated iron binding capacity; TIBC, indicates total iron-binding capacity; TS, transferrin saturation; WBCs, white blood cells; RBCs, red blood cells; Hb, hemoglobin; Hct, hematocrit; MCV, mean corpuscular volume; MCH, mean corpuscular hemoglobin, n = 20 for each group.

**Table S2 Body weight and plasma lipids of *Apoe^-/-^* and *Apoe^-/-^Fpn1^LysM/LysM^* mice.**

|  | **Body Weight (g)** | | |
| --- | --- | --- | --- |
|  | ***Apoe^-/-^*** | ***Apoe^-/-^Fpn1^LysM/LysM^*** | ***P*** |
| 8-week weight | 20.2±0.8 | 20.4±1.2 | > 0.05 |
| 24-week weight | 25.4±1.0 | 25.7±1.2 | > 0.05 |
| Gain Weight | 5.2±0.7 | 5.3±1.0 | > 0.05 |
| **Plasma lipids** |  | | |
| Total Cholesterol (mmol/L) | 17.15±1.31 | 17.28±1.55 | > 0.05 |
| Triglycerides (mmol/L) | 1.03±0.11 | 1.10±0.15 | > 0.05 |

Eight-weeks old mice were fed the Western diet for another 16 weeks. Blood was drawn (detail sees ***Blood samples and tissue collection***). Plasma lipids include cholesterol and triglyceride. Data are represented as mean ± SEM. Statistical significance was determined using Student’s *t*-test. No significant difference was found, n = 20 for each group.

**Table S3 Serum and hematologic parameters of *Apoe^-/-^Fpn1^LysM/LysM^* mice injected with saline or DFP.**

|  | **Saline Injection** | **DFP Injection** | ***P*** |
| --- | --- | --- | --- |
| **Serum parameters** | | | |
| Serum iron, μg/dL | 118±10 | 97±10 | < 0.01 |
| UIBC, μg/dL | 197±25 | 244±11 | < 0.001 |
| TIBC, μg/dL | 316±33 | 340±13 | < 0.05 |
| TS, % | 37.4±2.0 | 28.5±2.5 | < 0.001 |
| **Hematology** | | | |
| RBCs (x10^12^/L) | 9.2±0.8 | 7.5±1.6 | < 0.05 |
| Hb (g/L) | 115±15 | 86±14 | < 0.05 |
| Hct (%) | 34.8±3.1 | 22.9±5.4 | < 0.05 |
| MCV (fl) | 38.9±1.7 | 37.6±3.4 | > 0.05 |
| MCH (pg) | 13.5±0.8 | 11.1±1.3 | < 0.05 |

Eight-weeks old mice were fed the Western diet for another 16 weeks with DFP treatment (80 mg/kg). Saline was used as a vehicle. Blood was drawn (detail sees ***Blood samples and tissue collection***). Data are represented as mean ± SEM. Statistical significance was determined using Student’s *t*-test. The abbreviation is the same as in **Table S1**, n = 10 per group.

**Table S4 Body weight and plasma lipids of *Apoe^-/-^Fpn1^LysM/LysM^* mice injected with saline or DFP.**

|  | **Body Weight (g)** | | |
| --- | --- | --- | --- |
|  | **Saline Injection** | **DFP Injection** | ***P*** |
| 8-week weight | 20.8±1.5 | 21.0±2.0 | > 0.05 |
| 24-week weight | 25.9±1.4 | 25.5±1.8 | > 0.05 |
| Gain Weight | 5.1±2.3 | 4.5±2.7 | > 0.05 |
| **Plasma lipids** |  | | |
| Total Cholesterol (mmol/L) | 17.40±2.18 | 17.32±2.43 | > 0.05 |
| Triglycerides (mmol/L) | 1.07±0.13 | 1.16±0.18 | > 0.05 |

The mice were treated the same as in **Table S3**. Plasma lipids include cholesterol and triglyceride. Data are represented as mean ± SEM. Statistical significance was determined using Student’s *t*-test. No significant difference was found, n = 10 per group.

**Additional Methods**

**Blood samples and tissue collection**

The mice were anesthetized with an intraperitoneal injection of pentobarbital sodium (40 mg/kg) and euthanized by cervical dislocation. Blood was drawn from the inferior vena cava and collected in heparinized tubes. Plasma was prepared by centrifugation (1,200 × g) for 15 min at 4°C. Plasma samples were then stored at -80°C for determination of plasma cholesterol concentration and cytokine levels. The mice were then perfused with 4°C saline through the left ventricle. After perfusion, the arteries, hearts, livers and spleens were harvested. The samples were fixed in 4 % paraformaldehyde or quickly frozen at -80°C for further analysis.

**Determination of plasma cholesterol concentrations and cytokine levels**

Total cholesterol (TC) and triglyceride (TG) in plasma were determined by auto chemical analyzer (Beckman Coulter AU5421, CA). Hepcidin (Intrinsic Lifescience, La Jolla, CA), IL-6, IL-1β, TNF-α, MCP-1, and ICAM-1 (Elabscience Biotech Co, Ltd, Wuhan, China) were detected by ELISA assays according to the manufacturer's protocols.

**Lipid accumulation and lesion area in the aorta and the aortic root**

Lipid content were determined with Oil Red O to stain the aorta and aortic sinus sections. For assessment of atherosclerotic lesion area, the aorta was analyzed from aorta arch to abdominal aortic bifurcation. For assessment of atherosclerotic lesion size in aortic sinus, serial 6 μm thick cryosections from the middle portion of the ventricle to the aortic arch were collected. The quantification of lesion area and size was analyzed using Image J software.

**Immunohistochemical (IHC) and Masson's Trichrome staining**

Sections of aortic root were used to assess the plaque composition by Masson's Trichrome staining for collagen and IHC staining for CD68, smooth muscle actin (SMA), and 8-OHDG. These three primary antibodies were purchased from Abcam (1:200 dilution, Cambridge, MA), and the secondary antibodies from Santa Cruz Biotech (1:200, Santa Cruz, CA).

Images were captured under a light microscope (Zeiss, Germany). For quantitative analysis of images, 3 sections per animal at intervals of 30 μm were analyzed. The intensity of positive staining was analyzed by Image J software.

**Iron assays**

Deparaffinized tissue sections were stained with the Perls’ Prussian blue stain for nonheme iron as previously described[^1^](#_ENREF_1)^,^ [^2^](#_ENREF_2). Deparaffinized tissue sections were incubated for 30 min in 1% potassium ferrocyanide in 0.12 N HCl.

Total non-heme iron in the tissues was measured by using a colorimetric ferrozine-based assay as previously described[^3^](#_ENREF_3). Briefly, 22 μl concentrated HCl (11.6 M) was added to 100 μl of homogenized tissue samples (about 500 μg total protein). The sample was then heated at 95°C for 20 min, followed by centrifugation at 12,000 × g for 10 min. The supernatant was transferred into a clean tube. Ascorbate was added to reduce the Fe^3+^ into Fe^2+^. After 2 min of incubation at room temperature, ferrozine and saturate ammonium acetate (NH4Ac) were sequentially added to each tube and the absorbance was measured at 570 nm (BioTek ELx800, Shanghai, China) within 30 min.

**Western blotting**

Protein lysates were run in gels and transferred to membranes as previously reported[^4^](#_ENREF_4). The membranes were probed using antibody directed against CAT, SOD2, IL-1β, IL-6, ABCG1, ABCA1, and LOX-1 purchased from Abcam, TNF-α from Santa Cruz Biotech., HO-1, LXRα, CD36 from Proteintech Group Inc. (Chicago, IL), and GAPDH or actin from Bioworld Tech. (St. Louis Park, MN).

**Cholesterol content**

The content of lipids including total cholesterol (TC) and free cholesterol (FC) of ox-LDL-treated peritoneal macrophages were measured using enzymatic assay kits according to manufacturer instructions (Abcam). The conjugated cholesterol was calculated as cholesteryl ester (CE) using the following formula: CE = TC - FC.

**Cholesterol efflux assays**

Peritoneal macrophages were loaded with oxLDL (50 μg/ml) for 48 h. After the incubation, cells were washed twice in PBS and ApoAI-mediated cholesterol efflux studies were immediately performed by adding fresh RPMI medium without Nutridoma with or without 100 μg/ml of ApoAI (Sigma-Aldrich) for 24 h. In the case of experiments involving DFP or α-LA (Sigma-Aldrich), after cholesterol loading, *ApoE^-/-^Fpn1^LysM/LysM^* cells were placed in RPMI media with or without DFP or α-LA. Intracellular lipids were measured as described above. The percent change of intracellular cholesterol amounts in the presence of ApoAI relative to ApoAI-free medium was expressed according to the following equation: percent decrease in cellular cholesterol (%) = [1- (cellular cholesterol) ApoAI / (cellular cholesterol) ApoAI-free] × 100%.

**Quantitative real-time PCR (qRT-PCR)**

Total cellular RNA was isolated from peritoneal macrophages using Trizol (Invitrogen, Carlsbad, CA) and was reversely-transcribed to cDNA. qRT-PCR experiments were performed with SYBR Green PCR master mixture (Thermo Fisher Scientific). The primer sequences are as followed: 5'- GCTTGTTGGCCTCAGTTAAGG-3' and 5'- GTAGCTCAGGCGTACAGAGAT-3' for ABCA1, 5'- CTTTCCTACTCTGTACCCGAGG-3' and 5'- GGGGGCATTCCATTGATAAGG-3' for ABCG1, 5'-TGCACCACCAACTGCTTAGC-3' and 5'- GGCATGGACTGTGGTCATGAG - 3' for GAPDH.

**DHE staining**

ROS activity was evaluated in situ using DHE (10 mM, Sigma-Aldrich) staining. Briefly, fresh aortic sections were immediately embedded in Tissue-Tek OCT Compound (Sakura Finetek Japan, Tokyo, Japan) and cut into 6 μm thick sections and placed on glass slides. DHE was applied to each tissue section, and the slides were subsequently incubated at 37°C in the dark for 30 min. Images were immediately obtained with a fluorescence microscope (Axio Vert A1; Zeiss). For *ex vivo* assays, macrophages were washed and loaded with DHE (10 mM) for 30 min at 37°C and then images were captured by a fluorescence microscope.

**References**

1. Donovan A, Lima CA, Pinkus JL, Pinkus GS, Zon LI, Robine S, Andrews NC. The iron exporter ferroportin/Slc40a1 is essential for iron homeostasis. *Cell metabolism* 2005;**1**:191-200.

2. Wang Q, Ji J, Hao S, Zhang M, Li K, Qiao T. Iron Together with Lipid Downregulates Protein Levels of Ceruloplasmin in Macrophages Associated with Rapid Foam Cell Formation. *Journal of atherosclerosis and thrombosis* 2016;**23**:1201-1211.

3. Li H, Zhao H, Hao S, Shang L, Wu J, Song C, Meyron-Holtz EG, Qiao T, Li K. Iron regulatory protein deficiency compromises mitochondrial function in murine embryonic fibroblasts. *Scientific reports* 2018;**8**:5118.

4. Cai J, Jiang Y, Zhang M, Zhao H, Li H, Li K, Zhang X, Qiao T. Protective effects of mitochondrion-targeted peptide SS-31 against hind limb ischemia-reperfusion injury. *Journal of physiology and biochemistry* 2018;**74**:335-343.
